# Perimesencephalic Subarachnoid Hemorrhage After Thunderclap Headache in a Clinically Stable Patient: A Case Report

**DOI:** 10.7759/cureus.113042

**Published:** 2026-07-20

**Authors:** Amjad Ahmed, Nazreen Banu Unnialukkal

**Affiliations:** 1 Emergency Department, Withybush General Hospital, Carmarthen, GBR; 2 General Practice, Withybush General Hospital, Carmarthen, GBR

**Keywords:** ct angiography (cta), digital subtraction angiography (dsa), non-aneurysmal sah, perimesencephalic subarachnoid hemorrhage, subarachnoid hemorrhage (sah), thunderclap headache

## Abstract

Subarachnoid hemorrhage (SAH) is a serious neurological emergency with a typical presentation of a thunderclap headache. SAH is a cause of considerable morbidity and mortality, especially in aneurysmal form. Improvement or even resolution of symptoms does not exclude potentially dangerous intracranial pathology in this context, which often leads to delayed diagnosis. We describe a case of perimesencephalic SAH in a young male who experienced a thunderclap headache, after which he became clinically stable. Subsequent imaging confirmed perimesencephalic SAH, while the exclusion of a vascular lesion was established by CT angiography, digital subtraction angiography, and 3D rotational angiography. Thus, a thunderclap headache should be evaluated in every patient, irrespective of the dynamics of symptoms.

## Introduction

Subarachnoid hemorrhage (SAH) is a rare cause of headache seen in about 1-3% of cases, which nevertheless is very clinically significant due to a high mortality rate, which is 40-50% in aneurysmal SAH. Therefore, timely detection and diagnosis are necessary. Unfortunately, diagnosing SAH in a stable patient can become challenging since the improvement of symptoms makes the task even more complicated [1].

The sensitivity of non-contrast CT of the head in identifying SAH in a patient presenting within six hours of symptom onset reaches 98-100%. With increasing time after symptom onset, the sensitivity of CT drops to 85-90% in 24 hours due to increased difficulty in detecting blood in the subarachnoid space. Thus, the absence of blood in the brain's subarachnoid space seen in a patient with symptoms persisting for more than 24 hours means little and requires further examination through lumbar puncture or CT angiography [2].

Perimesencephalic subarachnoid hemorrhage (PMSAH) is known as a distinctive subtype of SAH and occurs in about 5-10% of all cases. The defining feature of PMSAH is the location of blood in cisterns in front of the brainstem and the midbrain, especially in the prepontine and interpeduncular cisterns. Contrary to aneurysmal SAH, the pathogenesis of PMSAH involves the rupture of small venous structures in the prepontine region, which is proven by the absence of any vascular lesions detected by imaging [3,4].

## Case presentation

A 41-year-old male with type 1 diabetes presented to the emergency department in good condition 24 hours after having a sudden occipital headache radiating to the neck, for which he was immediately referred to the hospital by his general practitioner. He described the severity of his headache as 8/10, without loss of consciousness, photophobia, nausea or vomiting, and other focal neurologic symptoms.

Symptoms had improved significantly in the last 24 hours, and only a mild persistent headache remained at the time of arrival, which worsened when sitting up. There was no preceding physical activity, sex, performance of the Valsalva maneuver, or trauma in the patient's history. He received subcutaneous insulin therapy due to type 1 diabetes with HbA1c at 6.5%. There were no anticoagulants or antiplatelet medications, and no positive history of family members with an intracranial aneurysm, polycystic kidney disease, or connective tissue disease.

Upon arrival, the patient was oriented, with a Glasgow Coma Scale score of 15/15 and stable vital signs: blood pressure of 134/72 mmHg, heart rate 76 beats per minute and regular, oxygen saturation of 98% on room air, and body temperature of 36.8°C. Neurological examination was entirely normal, with no weakness or sensory deficits, normal function of all cranial nerves, and normal cerebellar signs. There were no signs of meningism, with no neck stiffness or positive Kernig sign. Fundoscopy was not conducted since the history suggested a thunderclap headache and required imaging. Due to clinical characteristics, the diagnosis was suspected, and a decision was made to conduct further tests.

**Figure 1:**
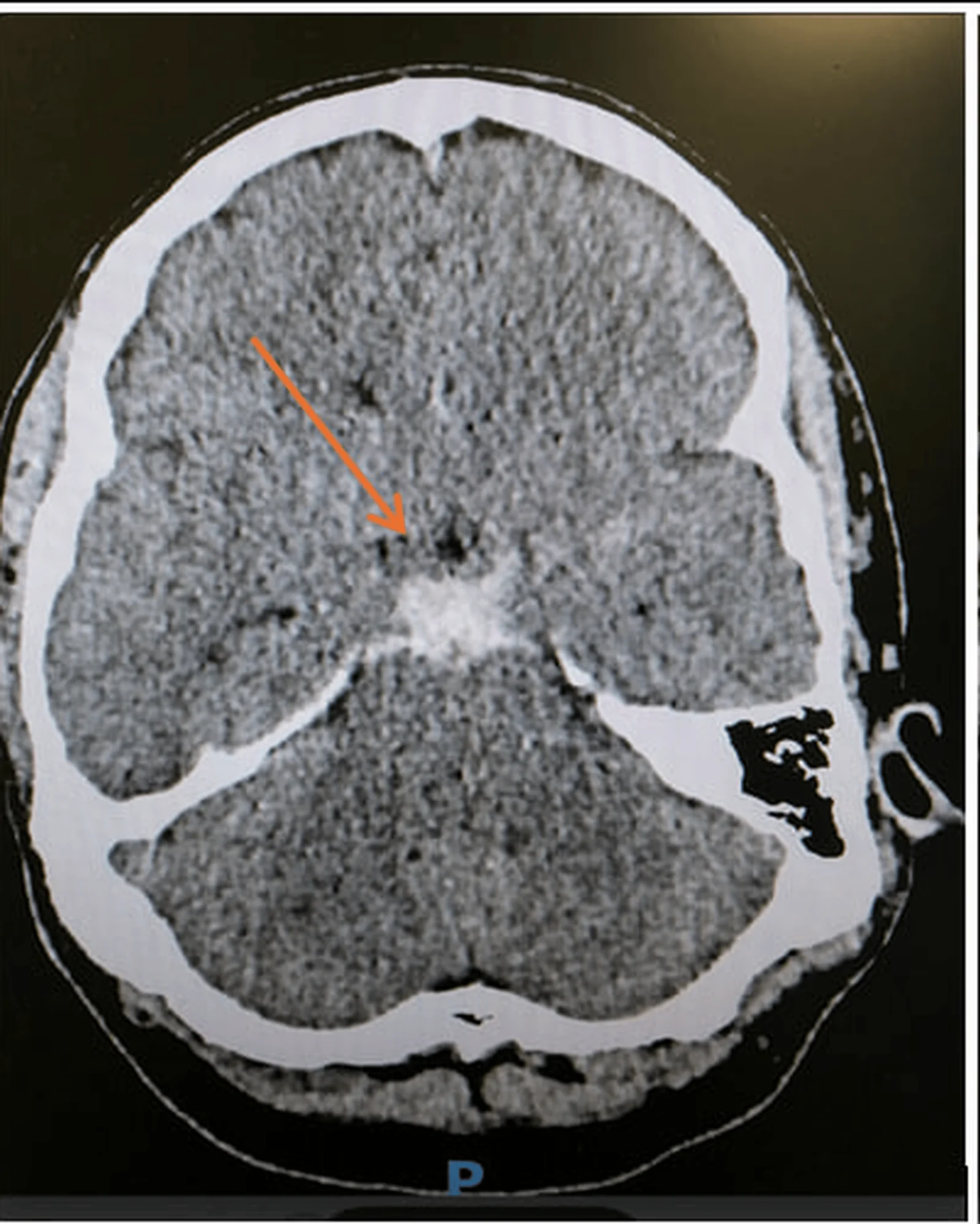
Non-contrast CT of the head showing perimesencephalic subarachnoid hemorrhage (PMSAH, arrow) confined to the prepontine and interpeduncular cisterns, consistent with the diagnosis of PMSAH.

In about 26 hours from symptom onset, a non-contrast CT of the head was performed and found SAH located strictly in the perimesencephalic cisterns, specifically in the prepontine and interpeduncular cisterns. Blood was localized strictly in these areas without extension to the Sylvian fissures, interhemispheric fissures, cerebral hemispheres' surfaces, ventricles, or hydrocephalus. Intracerebral hemorrhage was not identified. These features strongly suggested the diagnosis of perimesencephalic SAH (Figure 1).

**Figure 2:**
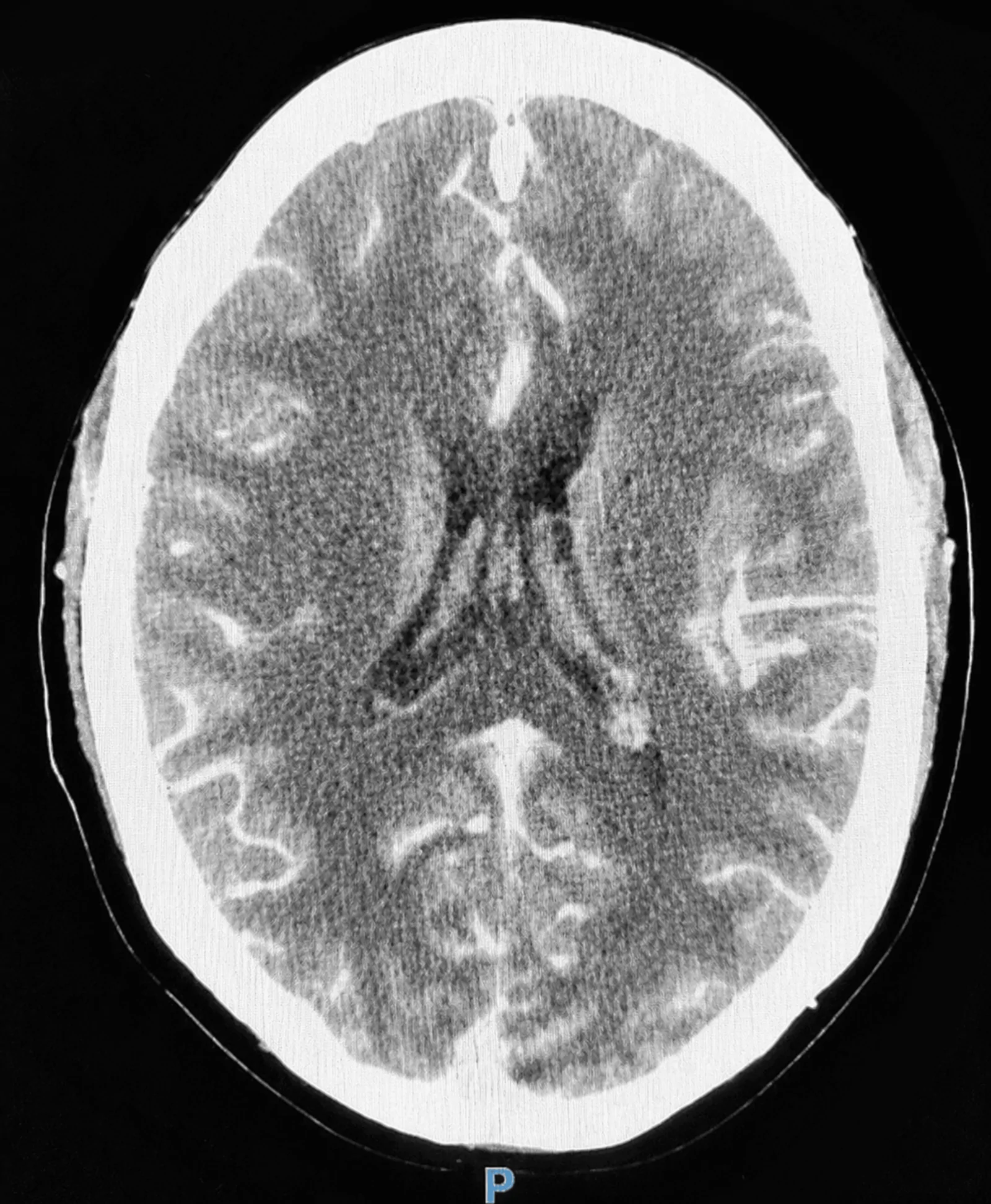
CT angiogram showing no intracranial aneurysm/arteriovenous malformation.

Since SAH was confirmed on CT imaging, further lumbar puncture was not necessary. Further CT angiography revealed no aneurysm, vascular malformation, or arterial dissection, which was the main aim of the study, and thus confirmed the suspicion of PMSAH (Figure 2).

Laboratory test results, including the complete blood count, coagulation profile, renal and liver function tests, and blood glucose level, were within the reference range. ECG did not reveal acute abnormalities.

Several diagnoses were considered, including an aneurysmal SAH, which was mainly suspected due to the patient's typical symptoms. PMSAH became more probable with imaging revealing a pattern typical of PMSAH and the absence of an aneurysm in vascular imaging. Cervical artery dissection was possible as well in the presence of severe headache and neck pain. However, no neurologic deficit was observed. Reversible cerebral vasoconstriction syndrome could also be diagnosed since it is one of the causes of thunderclap headache. Recurrent headaches were ruled out. The diagnosis of cerebral venous sinus thrombosis was considered unlikely as well, based on imaging findings. The diagnoses of idiopathic intracranial hypertension and intracranial hypotension were unlikely, except for postural headache, which raised the question.

Since SAH was suspected and imaging confirmed its presence, the patient was prescribed nimodipine 60 mg orally every four hours to minimize the risk of cerebral vasospasm. It is a routine practice when suspecting aneurysmal SAH to prevent the development of delayed cerebral ischemia. Neurological observations, including neurological examination, pupils' assessment, and blood pressure monitoring, were done frequently. Pain treatment was administered using only paracetamol since nonsteroidal anti-inflammatory drugs were contraindicated as they had antiplatelet properties in the current condition. Additionally, the patient was provided with instructions concerning activity restriction and fluid intake during recovery.

There were no signs of vasospasm, cerebral ischemia, hydrocephalus, or worsening neurological symptoms. Glucose levels remained in the target range. Further negative findings on vascular imaging resulted in a definitive diagnosis of non-aneurysmal PMSAH. The patient completed the nimodipine treatment course and left the hospital with recommendations for future safety netting. He was instructed to visit a neurologist upon discharge and contact his general practitioner in case of recurrent thunderclap headache.

## Discussion

This case highlights an important diagnostic challenge in emergency medicine: patients presenting with a classic thunderclap headache may appear clinically well by the time of assessment. Despite significant symptomatic improvement and a normal neurological examination, the initial history remained highly suspicious for SAH. This emphasizes that clinical improvement should never reassure clinicians sufficiently to exclude SAH without appropriate neuroimaging [5,6]. Diagnostic delays continue to occur, particularly in patients without meningism or persistent severe symptoms, increasing the risk of adverse outcomes [7,8].

In this patient, non-contrast CT performed approximately 26 hours after symptom onset demonstrated hemorrhage confined to the prepontine and interpeduncular cisterns without extension into the Sylvian fissures, interhemispheric fissure, ventricles, or cortical sulci, supporting a perimesencephalic hemorrhage pattern. Although CT sensitivity declines after six hours, blood remained clearly visible, allowing diagnosis without lumbar puncture [9]. Subsequent CT angiography, digital subtraction angiography, and three-dimensional rotational angiography demonstrated no aneurysm, vascular malformation, or arterial dissection, supporting the diagnosis of non-aneurysmal perimesencephalic SAH [10]. Dedicated cerebral venous imaging was not performed, and this limitation has been acknowledged because venous bleeding has been proposed as a possible mechanism in perimesencephalic SAH [11].

Perimesencephalic SAH represents approximately 5-10% of spontaneous SAH and is recognized as a distinct clinical and radiological entity with a substantially more favorable prognosis than aneurysmal SAH [12]. Accurate recognition of the hemorrhage distribution is essential because management, prognosis, and follow-up differ from aneurysmal hemorrhage. In carefully selected patients with a characteristic hemorrhage pattern and comprehensive negative vascular imaging, the diagnostic yield of repeat angiography is extremely low [13].

Management of our patient initially followed the standard pathway for suspected aneurysmal SAH until vascular causes had been excluded. He remained neurologically stable throughout admission, developed no vasospasm, hydrocephalus, or delayed cerebral ischemia, and achieved an uncomplicated recovery. His co-existing type 1 diabetes required careful glucose monitoring during admission because metabolic disturbances may adversely influence neurological recovery [13].

Overall, this case reinforces that thunderclap headache warrants urgent investigation irrespective of symptom resolution. Careful interpretation of hemorrhage distribution together with systematic vascular imaging is essential to distinguish non-aneurysmal perimesencephalic SAH from aneurysmal hemorrhage and other vascular causes. Once aneurysmal disease has been confidently excluded, patients with perimesencephalic SAH generally have an excellent prognosis and a favorable long-term outcome [13].

## Conclusions

This case illustrates several important issues in the diagnosis and management of SAH. A thunderclap headache should always be considered a serious neurological emergency and warrants hospital evaluation even if symptoms improve. The sensitivity of CT imaging decreases over time; therefore, a negative CT scan performed more than 24 hours after symptom onset does not exclude SAH, and additional diagnostic investigations should be undertaken. Vascular imaging should be performed systematically, with CT angiography serving as the first-line modality for identifying underlying vascular abnormalities. The prognosis of perimesencephalic SAH is generally excellent, with a very low risk of recurrence. Furthermore, in patients with diabetes, meticulous monitoring and control of blood glucose levels are essential throughout hospitalization to optimize outcomes and reduce complications.
